# Parathyroid carcinoma and pheochromocytoma in a patient with neurofibromatosis type 1: a rare association

**DOI:** 10.1530/EDM-24-0077

**Published:** 2025-02-10

**Authors:** Jardelina Brena Rocha Leite, Nicole Ramalho De Freitas, Rafaella Nelice de Holanda Cardoso, Erik Trovão Diniz, Gabriel Rodrigues de Assis Ferreira, Fernão Henrique Costa e Alvim, Camila Ribeiro Coutinho Madruga, Ana Carolina Thé Garrido, Luciano Albuquerque, Patricia Sampaio Gadelha, Ruy Lyra, Lucio Vilar

**Affiliations:** Universidade Federal de Pernambuco, Recife, Brazil

**Keywords:** endocrine cancers, rare diseases/syndromes, neuroendocrinology, adrenal, calcium

## Abstract

**Summary:**

The case report outlines a 33-year-old woman with neurofibromatosis type 1 (NF1) presenting complex symptomatology including a cervical mass, bone pain and significantly elevated calcium and parathyroid hormone levels, indicative of parathyroid carcinoma accompanied by cystic fibrous osteitis. Intriguingly, an incidental finding of an adrenal nodule prompted investigation, leading to the diagnosis of pheochromocytoma. Surgical intervention confirmed the presence of pheochromocytoma and parathyroid carcinoma. Genetic analysis corroborated NF1 with a pathogenic variant in the *NF1* gene. The patient’s clinical manifestations, coupled with the presence of café-au-lait spots and axillary freckles, supported the diagnosis of NF1. This case not only highlights the challenging diagnostic landscape of NF1 but also underscores the rarity of the co-occurrence of parathyroid carcinoma and pheochromocytoma within the context of NF1. It emphasizes the necessity for heightened clinical suspicion and comprehensive evaluation in patients with NF1, particularly in those presenting with endocrine abnormalities. Further investigation into the underlying mechanisms linking these conditions is warranted to elucidate their pathophysiological interplay and inform optimal therapeutic strategies for affected individuals. This case underscores the importance of multidisciplinary collaboration in the management of complex NF1-associated manifestations, with an emphasis on early detection and tailored intervention to optimize patient outcomes.

**Learning points:**

- Rare but real: multiple endocrine tumors can coexist in patients with neurofibromatosis type 1 (NF1), not only pheochromocytoma.
- Early detection matters: prompt diagnosis and a multidisciplinary approach are crucial for managing NF1 patients presenting with symptoms suggestive of these rare endocrine tumors.
- Genetic insights guide care: genetic testing aids in confirming NF1 and guiding treatment decisions, emphasizing the role of genetics in personalized medicine for NF1 patients.

## Background

Neurofibromatosis type 1 (NF1) is an inherited disorder that primarily affects the central nervous system and skin. Individuals with NF1 have a significantly higher risk of developing both benign and malignant tumors, with such tumors occurring up to four times more frequently than in the general population (1). A variety of endocrine tumors have been linked to NF1, with pheochromocytoma being the most common. The prevalence of pheochromocytoma in NF1 patients ranges from 0.1 to 5.7% (2). A recent monocentric study screening 108 NF1 patients identified pheochromocytoma in 24 (22.2%) of them (3). Furthermore, a large retrospective study of 1,415 patients found pheochromocytoma in 41 (2.9%) cases (4). Other endocrine abnormalities associated with NF1 include follicular thyroid adenoma (1.4%), parathyroid gland adenoma (1.4%) and C-cell hyperplasia of the thyroid gland (1.4%) (2). NF1 is also linked to various other endocrine disorders, such as delayed or premature puberty due to optic pathway gliomas (5), prepubertal gynecomastia (6) and low bone density, which increases the risk of fractures by three to five times (7). Although the association between NF1 and primary hyperparathyroidism has been described, it is considered a very rare condition, especially when it involves parathyroid carcinoma (1). In this report, we describe a case of parathyroid carcinoma combined with pheochromocytoma in a patient with NF1, with the diagnosis confirmed by genetic mutation in the *NF1 *gene.

## Case presentation

A 33-year-old woman was referred to our outpatient clinic with pain and swelling in the left anterior cervical region for two years. She also complained of generalized bone pain, which had prevented her from walking for four months. In addition, she presented with generalized muscle weakness, both proximal and distal, likely due to chronic immobility. She denied comorbidities, except for bilateral deafness since childhood. On physical examination, the cervical mass was visible, approximately 5 cm long, had a hard consistency and little to no mobility. Brownish stains distributed over the abdomen and thighs and freckles in the armpits were also visible. No Lisch nodules, neurofibromas or postural blood pressure drops were detected during the assessment.

## Investigation

**Figure 1:**
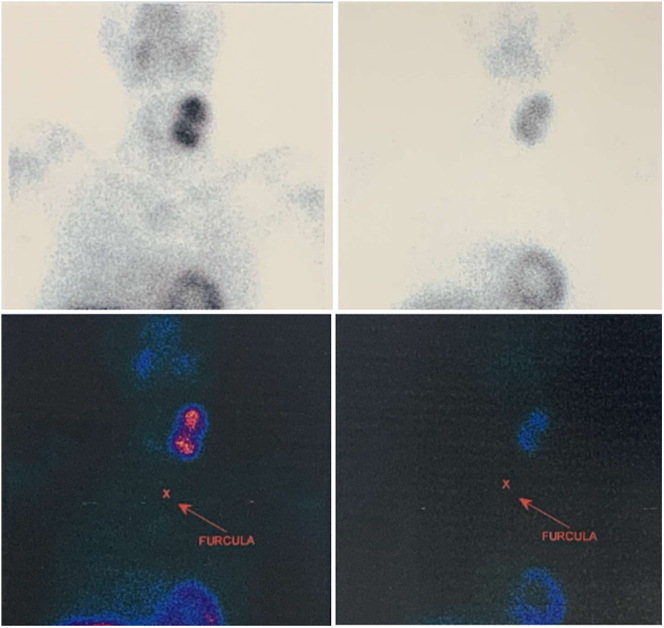
Parathyroid scintigraphy with SESTAMIB with a large area of tracer uptake in the projection of the left lobe of the thyroid (on the left), with significant retention in the late images (on the right).

The patient underwent laboratory tests revealing serum total calcium of 19.3 mg/dL, parathyroid hormone (PTH) of 2,462 pg/mL and 25-OH-vitamin D of 13 ng/mL (Table 1). The radiographic bone studies revealed multiple lytic lesions in the skull and long bones, compatible with cystic fibrous osteitis. Due to hypercalcemia, electrocardiogram (ECG) was performed, and no abnormalities were detected. Cervical ultrasonography revealed a nodule on the left side, measuring 5 cm in its largest diameter, suggestive of a parathyroid nodule. Parathyroid scintigraphy and whole-body scanning with SESTAMIB pointed to a large area of pronounced and heterogeneous uptake of the tracer in the projection of the left thyroid lobe, with significant retention in late images, in addition to a diffuse and heterogeneous increase in tracer uptake in practically the entire skeleton, suggestive of brown tumors (Fig. 1). The patient underwent bone densitometry, which revealed low bone mass for age, with a *Z*-score of −5.0 in the distal radius, and an abdominal computed tomography (CT) showing non-obstructive microcalculi in the left kidney.

**Table 1:** Laboratorial test results.

Test	Results	Reference range
Serum calcium (mg/dL)	19.8	8.4–10.2
Serum albumin (g/dL)	4.04	3.5–4.8
Serum phosphorus (mg/dL)	4.2	3.4–4.7
Serum 25-OH-vitamin D (ng/mL)	13.8	≥30
Serum PTH (pg/mL)	2,462	15.0–68.3
Serum alkaline phosphatase (IU/mL)	263	40–150
Serum urea (mg/dL)	69	10–50
Serum creatinine (mg/dL)	1.85	0.6–1.3
Total serum metanephrine (pg/mL)	326	≤205
Serum normetanephrine (pg/mL)	179	≤148
Serum metanephrine (pg/mL)	147	≤57
Urinary normetanephrine (μg/24 h)	661.1	≤732
Urinary metanephrine (μg/24 h)	394.5	≤280
Chromogranin A (μg/L)	14	<100
Serum calcitonin (pg/mL)	<2.0	<5.0

**Figure 2:**
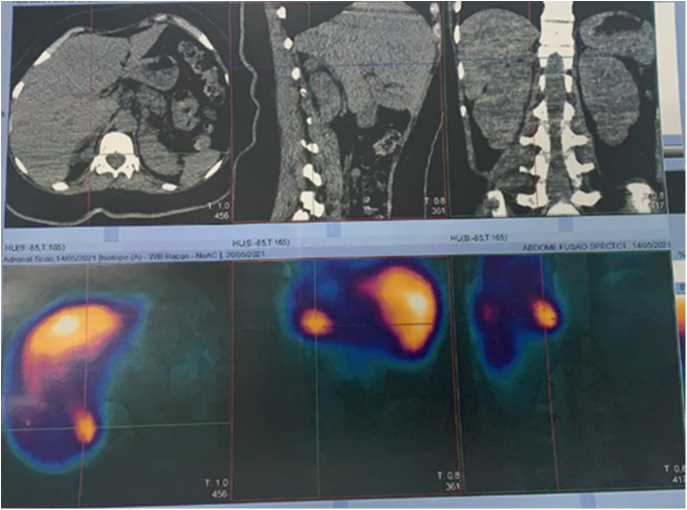
Abdominal single photon emission computed tomography with metaiodobenzylguanidine (MIBG) combined with computer tomography (SPECT-CT). We see increased radiotracer uptake in the topography of the right adrenal gland. From right to left, axial, lateral and coronal images.

Due to flu-like symptoms upon admission during the COVID-19 pandemic, a chest CT was performed, which incidentally detected a 2.0 × 1.3cm nodule on the right adrenal gland, with an attenuation coefficient of approximately 23 Hounsfield units. The patient had no previous diagnosis of hypertension and no arterial pressure peaks was caught during hospitalization. To evaluate the adrenal incidentaloma, serum and urinary metanephrines were measured using liquid chromatography–mass spectrometry, with a slight increase in their values (Table 1), and a whole-body scintigraphy with metaiodobenzylguanidine was performed, which was suggestive of a tumor of neuroendocrine origin in the right adrenal gland (Fig. 2).

The patient then underwent a right adrenalectomy and later, left parathyroidectomy and partial thyroidectomy. During the postoperative period, the patient experienced a slow and progressive drop in calcium levels, prompting the initiation of oral calcium and calcitriol supplementation. Histopathological analysis confirmed the diagnosis of pheochromocytoma, with a PASS score of 2, characteristic of benignity (PASS < 4). Immunohistochemical studies of the lesion were positive for chromogranin, synaptophysin and S-100, with a Ki-67 index of 1–2%.

**Figure 3:**
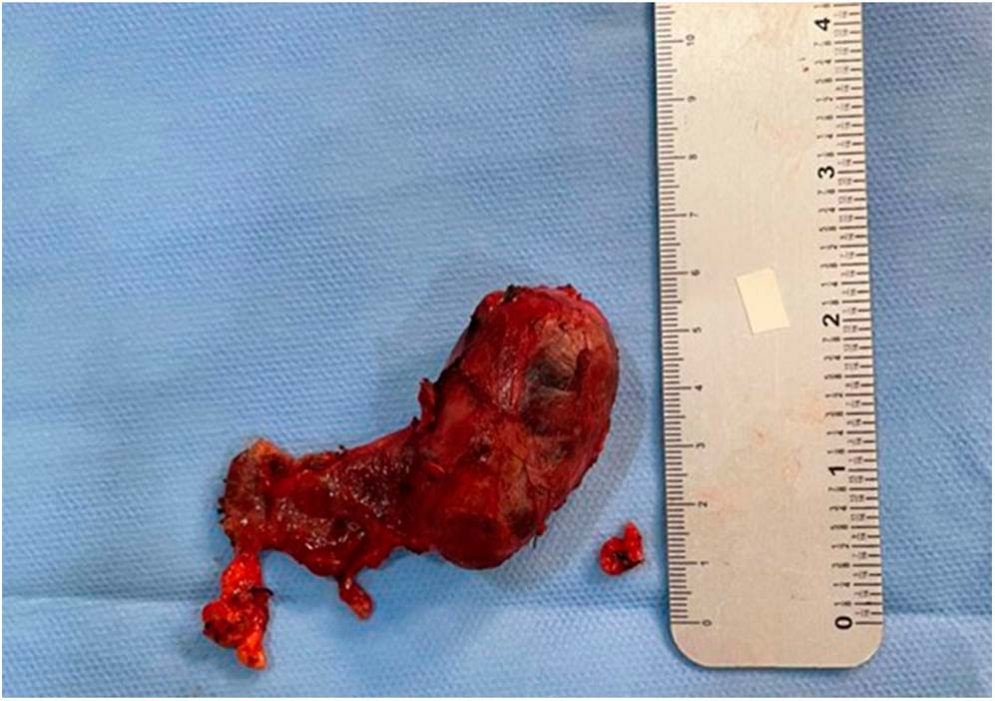
Cervical tumor resection product, measuring 7.0 × 5.5 × 4.0 cm. The carcinoma itself measured 4.8 × 2.8 × 2.4 cm.

The cervical tumor was identified as a parathyroid carcinoma, with a Ki-67 index of 2%. The surgical specimen measured 7.0 × 5.5 × 4.0 cm (Fig. 3), while the carcinoma itself measured 4.8 × 2.8 × 2.4 cm. Features indicative of malignancy included vascular invasion and infiltration of the tumor capsule. Despite these findings, there was no evidence of tumor necrosis, nuclear atypia or macronucleoli. The thyroid tissue sample was histologically normal, with no pathological abnormalities.

Faced with skin changes on physical examination, the patient was evaluated by our dermatology team, which confirmed findings compatible with café-au-lait spots suggestive of neurofibromatosis. For the investigation of hearing deficit, she underwent audiometry, which found bilateral sensorineural deafness, and magnetic resonance imaging of the temporal bones, which showed no alterations. A genetic test was then performed on suspicion of neurofibromatosis, confirming a heterozygous pathogenic variant in the *NF1 *gene (Gly629Arg variant).

## Treatment

During the postoperative period, the patient had a slow and progressive drop in calcium levels, and oral intake of calcium, calcitriol and colecalciferol were initiated. The doses were adjusted according to serum calcium, phosphate and 25-OH-vitamin D levels, and nowadays, she is using calcium carbonate 600 mg three times a day, calcitriol 0.25 μg once daily and colecalciferol 15,000 IU once weekly. For the parathyroid carcinoma, she did 30 sections of adjuvant radiotherapy.

## Outcome and follow-up

The patient is being followed up at our outpatient clinic three times a year, reporting relief from diffuse bone pain. She is also undergoing physical therapy for motor rehabilitation. After two years, the oncology team is now assessing the patient once a year. She has not developed hypoparathyroidism, shows no laboratorial evidence of pheocromocytoma (Table 2) and has increased hip *Z*-score to +0.9.

**Table 2:** Follow-up test results two years later (2023).

Test	Results	Reference range
Serum calcium (mg/dL)	9.7	8.4–10.2
Serum albumin (g/dL)	4.7	3.5–4.8
Serum phosphorus (mg/dL)	3.1	2.5–4.5
Serum 25-OH-vitamin D (ng/mL)	28.7	≥30
Serum PTH (pg/mL)	47.4	15.0–68.3
Serum alkaline phosphatase (IU/mL)	62	40–150
Serum urea (mg/dL)	76	10–50
Serum creatinine (mg/dL)	1.5	0.6–1.3
Urinary normetanephrine (μg/24 h)	173	≤732
Urinary metanephrine (μg/24 h)	<20	≤280

PTH, parathyroid hormone.

## Discussion

Neurofibromatosis is an autosomal dominant inherited multisystem neurocutaneous disorder that affects 1 in 2,700–3,000 live births. It is divided into neurofibromatosis type 1 (NF1), neurofibromatosis type 2 and schwannomatosis. NF1, the most common among them, is caused by a heterozygous germline mutation in the *NF1 *gene, located on chromosome 17q11.2 (8). This gene encodes the protein neurofibromin, responsible for the negative regulation of the RAS proto-oncogene, a key signaling molecule in the control of cell growth (9). The loss of its function can cause uncontrolled cell growth and formation of benign and malignant tumors (10). It is diagnosed clinically when two or more of the following seven features are identified: six or more café-au-lait spots, two or more neurofibromas, axillary or inguinal freckles, optic pathway glioma, two or more Lisch nodules, characteristic bone lesions and first-degree relative with neurofibromatosis (8). The patient described in this report has café-au-lait spots and axillary freckles, with no other characteristic findings of NF1. However, the association of two endocrine tumors pointed us to this diagnostic possibility, supporting genetic research.

Fewer than 1% of cases of hyperparathyroidism are caused by parathyroid carcinoma, one of the rarest malignant neoplasms, with an estimated prevalence of 0.005% of all cancers (11). It has a clinical presentation similar to benign parathyroid diseases, however, with generally higher levels of calcium and PTH, as reported in our patient. In 40–70% of the cases, there is a palpable neck mass, which was also detected in the physical examination of our patient, in addition to lymph nodes involvement that may be present in 15–30% of the cases at presentation and distant metastases that occur in approximately 30% of the patients, both of which were absent in our patient. With similar prevalence between genders, parathyroid carcinoma typically occurs between 45 and 59 years of age; however, we report an early presentation of the disease with diagnosis at 33 years of age (12).

A possible explanation for the association between the two diseases could be due to the embryological origin, since NF1 is considered a disease of the neural crest, which gives rise to parathyroid cells (13). However, studies carried out in patients with NF1 found no association with parathyroid carcinoma and few reports of the occurrence of hyperparathyroidism in these patients (14, 15). In a systematic review published in 2019, only 32 cases of hyperparathyroidism were found in patients with NF1, with only two cases of parathyroid carcinoma (16). For this reason, the association between NF1 and hyperparathyroidism in patients without multiple endocrine neoplasia types 1 and 2A should be considered as a coexisting condition rather than a clinical syndrome.

In most cases, pheochromocytoma is a benign tumor (90%), unilateral, sporadic and appearing in adulthood. When pheochromocytoma is familial, it usually occurs in isolation, but in 10% of cases it may be associated with syndromic genetic disorders such as multiple endocrine neoplasia type 2, von Hippel–Lindau disease and neurofibromatosis type 1. NF1 was the first gene identified as responsible for a genetic disorder associated with pheochromocytoma. Although there are few studies detailing specific features of pheochromocytomas in patients with neurofibromatosis, a review of multiple series by Walther *et al.* (17) in 1999 revealed that, with the exception of a predominantly unilateral presentation (more than 80% of cases), there do not seem to be any other peculiarities in relation to clinical presentation, diagnosis, evolution or treatment that allow differentially identifying pheochromocytomas associated with neurofibromatosis from those that develop into other hereditary syndromes (18).

Pheochromocytoma in NF1 can be completely asymptomatic and present as an adrenal incidentaloma (19). Therefore, such findings in imaging examinations should be valued in patients with suspected or confirmed NF1, as exemplified in our patient. However, the association between NF1 and hyperparathyroidism is not well-established, with very few cases described in the literature and no previously reported cases of simultaneous occurrence of pheochromocytoma and parathyroid carcinoma (16, 20). Parathyroid carcinoma, although extremely rare, is typically more aggressive than adenomas, with a higher recurrence rate and worse prognosis (12). Differentiating carcinoma from adenoma is crucial for guiding both surgical and long-term management strategies. This case highlights the importance of recognizing endocrine manifestations in NF1, as timely diagnosis and treatment of conditions such as pheochromocytoma and parathyroid carcinoma are critical to improving patient outcomes. The need for vigilant follow-up in cases of parathyroid carcinoma cannot be overstated, given its potential for recurrence and associated complications. This report further underscores the value of multidisciplinary care and genetic studies in complex presentations of NF1.

## Declaration of interest

The authors declare that there is no conflict of interest that could be perceived as prejudicing the impartiality of the work.

## Funding

We acknowledge the Tseu Medical Institute, Harris Manchester Collegehttps://doi.org/10.13039/100010370, Oxford, UK for research funding.

## Patient consent

Written informed consent was obtained from the patient for the publication of this case report. We also got approval of the Research Committee of our Service ‘Comitê de Ensino e Pesquisa (CEP)’.

## Author contribution statement

JBRL Leite, NR de Freitas and RN de Holanda Cardoso were the resident doctors who mainly assisted the patient during the investigation and took a major part in the writing of this report. ET Diniz is the main medical doctor (MD) who directly assisted the resident doctors. GR de Assis Ferreira, CRC Madruga and FH Costa e Alvim were resident doctors who assisted the patient during the follow-up. ACT Garrido, L Albuquerque, PS Gadelha, R Lyra and L Vilar are MDs of the Endocrinology team of our service who were all always present during case discussion and assessment of the patient. All authors contributed directly to patient caretaking. All authors reviewed and approved the final draft.

## Patient’s perspective

(Translated from Portuguese): I was in bed for months and I cannot describe how thankful I am for being able to walk normally again. My pain is almost gone and I can live my life once more. I am deeply grateful for the medical team and the hospital for everything they have done to me.
